# Home Hospitals Association

**Published:** 1902-03-22

**Authors:** 


### HOME HOSPITALS ASSOCIATION.

### THE UNDERLYING PRINCIPLE.

A bright little meeting took place on Tuesday, the 18th, at Fitzroy House, when a roomful of friends and supporters, as well as several medical men connected with the hospital, assembled to pass the annual report and transact other business. It is not often that the nursing staff is represented at hospital annual meetings, but the custom prevails at Fitzroy House, and the reception of visitors by the lady superintendent, the presence of several other ladies, and the generally "furbished up" appearance of the room, which was decorated with spring flowers, helped to make the business go pleasantly.

The chair was taken by Sir Henry Burdett, K.C.B., who, after referring to the year just closed, which had been an unusually successful one, devoted himself to the exposition of the principles on which, 25 years ago, the hospital was established, and which he would like to see extended, in the form of paying wards, to all the chief hospitals of London.

#### The Class of Patient.

In pleading for the necessary means (£7,000) to carry out the improvements referred to, the Chairman said that one of the misfortunes of money was that it was sometimes often accompanied by the inability to use it properly. By subscribing to the Home Hospitals Association a return was readily obtained in the assurance that alleviation of suffering was secured to a large class of persons who, though not poor, had but a narrow margin between income and expenditure. The existence of the hospital was due in a large measure to the interest taken in it by the Metropolitan Press, and he hoped that through their efforts the necessary sum would be raised for the alterations found to be necessary; 25 years ago Mr. John Walter, editor of the *Times*, and also the *Standard*, were very zealous in the matter, while the *Pall Mall Gazette* took upon itself to dispose of criticisms as they arose. He thought the home hospital had shown that it was fulfilling a useful work, and the Metropolitan Press might therefore let it be known that further help was now required. If the facts were made known, he had sufficient faith in the liberality of the public to believe that before very long the whole of the money would be readily forthcoming.

#### The Principle Involved.

"Adequate relief for all classes of the community who are sick, free from all possibility of abuse"—this he ventured to think should be the underlying principle of hospital administration. Why should hospitals only be for the poor? asked the *Times* in 1877, and not for all classes. That question had been continuously asked during the interval, and although it had been asked and answered in other countries, in England it remained unanswered. Three great interests were involved, the first of which was represented by the patients. Largely as a result of the Education Bill introduced and carried through by Mr. Forster, the class above the pauper class felt that they were not being justly treated in the great cities in times of illness. Why should they run the risks of treatment in their own homes, even with the aid of the district nurse, when they were willing to pay part of the cost of being treated in hospital? Whilst retaining their self-respect and independence, they claimed that they had a right to hospital treatment. In other countries, especially in the North of Europe, no class of patient was admitted to the hospital without being paid for, if not by himself, by the commune or municipality. In Sweden, for example, there were four grades of payment by which everybody could be treated without forfeiting either self-respect or independence. If he were asked how it was that the example set by these countries had not been followed, he would answer that it was because of the rooted objections of medical men and the managers generally to the system of payment. Such objections were based upon two main grounds. The medical practitioners considered it unjust to have paying patients introduced into the hospitals because it took away from them part of their means of livelihood. His answer was that no system of payment by patients would be adequate to meet the requirements of the patients unless it rendered such an objection impossible in practice. It was quite possible to provide that the paying wings should be absolutely distinct from the rest of each hospital, and the entrance could also be distinct. He believed that an arrangement of that kind with a percentage of the patients' payments set aside to pay the medical fees and the right for each patient to be attended by his own doctor would meet these objections. Hospital managers were lukewarm for financial reasons. They held that the public would say they had no assurance that their money was used to benefit the poor. But the suggestion of a paying wing, kept distinct, and with its own medical service, would largely remove that objection; and if the financial difficulty was still urged, he would point out that the giving of money was every year conducted on more intelligent lines, that donors of large sums had a clearer idea of what they wanted to support, and that they would give their money to those hospitals that were marked by the greatest intelligence in their management.

#### An Example from the United States.

The Johns Hopkins Hospital at Baltimore was one that everyone interested in hospital administration should see. The founder left directions that his money should be devoted to the purpose of a hospital in which a limited number of paying patients should be received. The trustees were thus enabled to provide for strangers, and those who, though friendless, were yet not objects of charity. In his opinion, any London hospital following the example of Baltimore would find its financial position strengthened, and it would be easier to raise money for its maintenance. It was at any rate perfectly certain that as population, and consequently demands, increased, unless there was a modification of the present system in the direction of paying wards, hospital abuses would continue to multiply, and the idea that free medical treatment was everybody's birthright would continue to grow.

An Example from a Poor District in London.

That it was possible to administrate a hospital on the lines he had indicated was proved by the Bolingbroke Hospital, in Battersea, where, in a very poor district, the medical profession were co-operating with the managers to work the hospital on a graduated system of payment by patients. Battersea had led the way in establishing a municipal ambulance department, and it was leading the way in hospital administration also. He believed the time had arrived when the people of London ought to awake, and, taking their courage in both hands, determine to provide hospitals with Pay Wings. It might be thought that he had departed from the object of the meeting, but those who were familiar with the articles of the association knew that, apart from its beneficent objects lay the principle that we ought not, in this country, to give free relief to people, unless they were entitled to receive it, but to provide hospital accommodation, and to afford to all patients an opportunity to retain the independence of manhood and womanhood. He would not have done his duty if he had not driven home the principles underlying the establishment of the Home Hospitals Association.

